# Critical Illness Secondary to Synthetic Cannabinoid Ingestion

**DOI:** 10.1001/jamanetworkopen.2020.8516

**Published:** 2020-07-20

**Authors:** Ismini Kourouni, Bashar Mourad, Hassan Khouli, Janet M. Shapiro, Joseph P. Mathew

**Affiliations:** 1 Department of Medicine, Mount Sinai Morningside and Mount Sinai West Hospitals, Icahn School of Medicine at Mount Sinai, New York, New York; 2 Now at Division of Pulmonary, Critical Care, and Sleep Medicine, MetroHealth Hospital Center, Case Western Reserve University, Cleveland, Ohio; 3 Now at Division of Pulmonary, Critical Care, and Sleep Medicine, New York University, New York, New York; 4 Now at Department of Critical Care Medicine, Cleveland Clinic, Cleveland, Ohio; 5 Now at Division of Pulmonary, Critical Care, and Sleep Medicine, Mount Sinai Morningside and Mount Sinai West Hospitals, Icahn School of Medicine at Mount Sinai, New York, New York

## Abstract

**Importance:**

Synthetic cannabinoids (SCs), commonly known as *K2*, *spice*, or *fake weed*, are cheap, artificially manufactured recreational drugs that have emerged as a major public health threat in various regions of the US.

**Objective:**

To describe the clinical manifestations of SC intoxication.

**Design, Setting, and Participants:**

This case series assessed adults admitted to the intensive care unit from 2014 to 2016 with acute life-threatening complications of SC use. Data analysis was completed in October 2016.

**Exposures:**

Use of SCs such as K2, spice, or other synthetic versions of cannabinoids.

**Main Outcomes and Measures:**

Data collected included patient demographic data, medical history, presenting symptoms, physical findings, laboratory and imaging data, and intensive care unit and hospital course.

**Results:**

Thirty patients (mean age, 41 years [range, 21-59 years]; 24 men [80%]) with SC ingestion were admitted to the intensive care unit over a 2-year period. Thirteen patients were undomiciled. The majority had a history of polysubstance abuse, psychiatric illness, or personality disorder. The admission diagnoses were coma (10 patients [33%]), agitation (10 patients [33%]), and seizure (6 patients [20%]). Eighteen patients (60%) had acute respiratory failure, and tracheal intubation was required in 21 patients (70%) for either airway protection or acute respiratory failure. Rhabdomyolysis was noted in 8 patients (26%). A man developed transient cerebral edema with loss of gray-white differentiation but had complete recovery. A woman with history of asthma died of acute respiratory distress syndrome. All patients underwent routine toxicology testing, which was unrevealing in 16 cases and revealed coingestion in the remainder. Sixteen patients (53%) left the hospital against medical advice.

**Conclusions and Relevance:**

Ingestion of SCs can lead to life-threatening complications, including severe toxic encephalopathy, acute respiratory failure, and death. Synthetic cannabinoids are undetectable in routine serum and urine toxicology testing but can be suspected on the basis of history and clinical presentation, which may include extreme agitation or coma. Frontline clinicians must be aware of the presentation and be vigilant in suspecting SC intoxication.

## Key Points

**Question:**

What are the clinical manifestations of synthetic cannabinoid intoxication, and are they life threatening?

**Findings:**

This case series of 30 adult patients describes the acute neurologic and cardiopulmonary complications of synthetic cannabinoid intoxication, including severe toxic encephalopathy, acute respiratory failure, and death.

**Meaning:**

These findings suggest that synthetic cannabinoids are a continued public health threat, with potential for morbidity and mortality from acute intoxication.

## Teaser

This case series describes the neurologic and cardiopulmonary complications associated with synthetic cannabinoid intoxication experienced by adult patients admitted to intensive care.

## Introduction

Synthetic cannabinoids (SCs), also known as *K2*, *spice*, and *fake weed*, are cheap, artificially manufactured recreational drugs that have emerged as a major public health threat in various regions of the world. Data from European, Australian, and American toxicology centers show a steady increase in SC use since 2012.^1,2^ On the basis of a preliminary report by the American Association of Poison Control Centers,^3^ at least 2695 exposures were confirmed in 2016 alone.

Our current knowledge of the physiologic effects of SC comes from case reports, toxicology case series, and forensic literature.^4,5,6,7^ More recent studies^8,9,10^ have shown that some synthetic cannabinoids are at least as potent as Δ9-tetrahydrocannabinol, whereas others can be as much as 100-fold more potent. There is no known antidote for SC intoxication.^11^ Clinical manifestations are unpredictable and vary by the type and the amount of SC used.^5,7^ Synthetic cannabinoids have neuropsychological effects due to the nonstandardized ingredients during production, and the toxic effects can vary widely.^12^ Producing SCs involves spraying different chemical compounds on various inert plant materials that are consumed with smoking.^10^ Furthermore, the Centers for Disease Control and Prevention reported the potential for addiction along with signs of withdrawal in regular users.^13,14^ The long-term effects of SC use are largely unknown.^6,15^

New York, New York, is a major trafficking center for SC, with episodes of mass intoxication occurring in 2015 and 2016.^16^ The increase in acute SC poisonings, notably with the substance K2, is the focus of our case series. We describe 30 patients with acute life-threatening neurologic and respiratory complications who required intensive care unit (ICU) admission within a 2-year period. To our knowledge, this is the largest reported series of critically ill patients with SC intoxication.

## Methods

 This case series received exempt approval from the institutional review board of Mount Sinai Morningside and Mount Sinai West Hospitals. A waiver of documented consent was granted by the institutional review board committees because this research presented no more than minimal risk of harm to participants. This study follows the Strengthening the Reporting of Observational Studies in Epidemiology (STROBE) reporting guideline.

We retrospectively reviewed records of 42 adult patients (aged ≥18 years) with reported SC intoxication who were admitted to the ICU at Mount Sinai Morningside and Mount Sinai West Hospitals during a 2-year period (2014-2016). Data were extracted from the medical record and prehospital records. All data were deidentified.

Patients were identified through ICU census records. The inclusion criterion was use of SC, as confirmed by either oral report from the patients or reported by bystanders or paramedics and documented in the medical record. Patients who denied use of SC, had high serum alcohol levels, or had urine toxicology studies that were positive for phencyclidine, amphetaminoids, or cocaine were excluded unless they reported such use more than 3 days before admission.

### Statistical Analysis

Data collected included demographic data, medical history, presenting symptoms, physical examination findings, laboratory and imaging data, need for intubation, ICU treatments, ICU and hospital length of stay, and outcome. A standardized worksheet was used for data abstraction. Data were collected from April 2016 to October 2016. Data analysis was completed in October 2016 using Excel software version 16 (Microsoft Corp).

## Results

**Figure 1.**
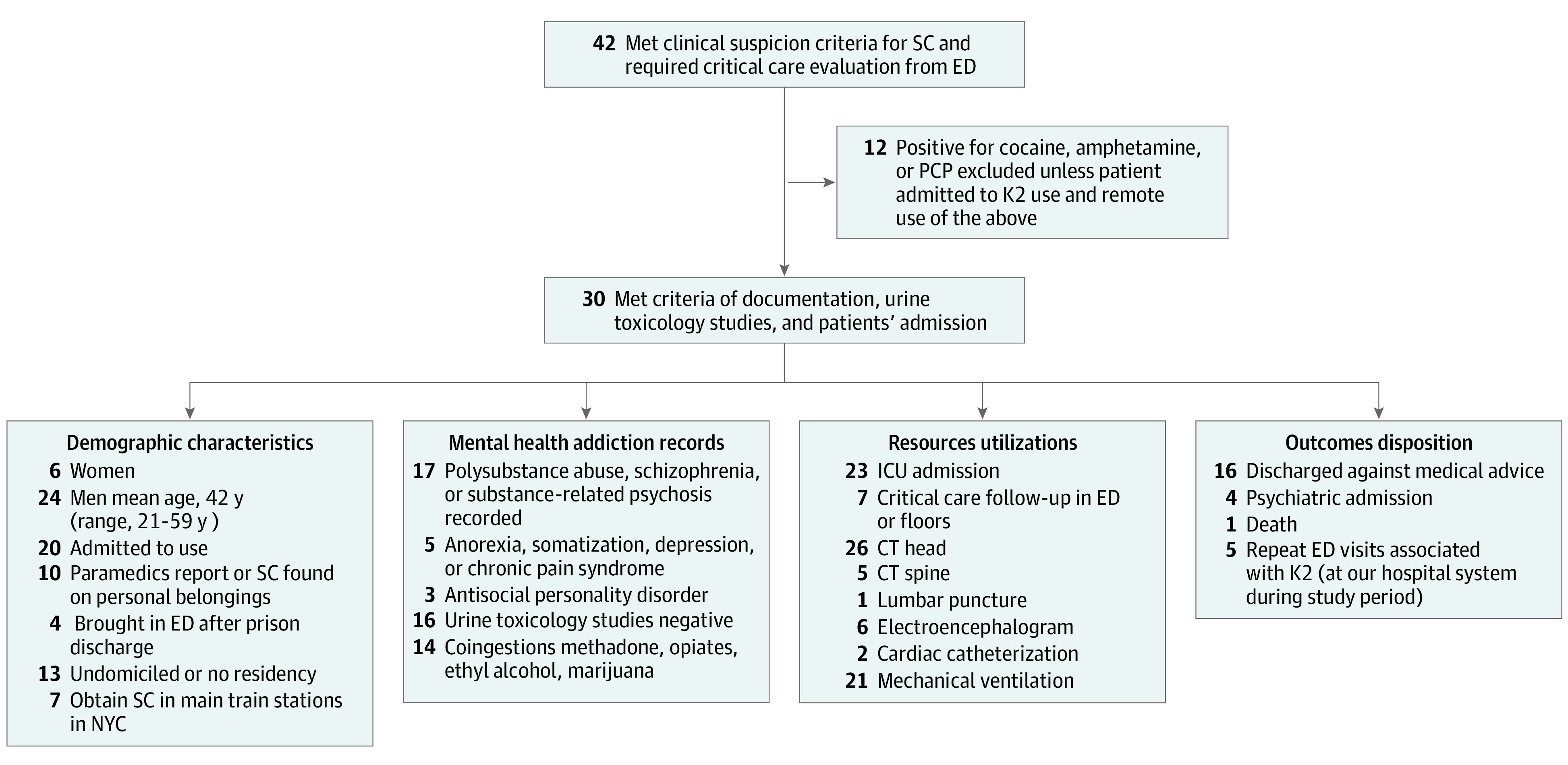
Participant Recruitment Flowchart Chart shows synthetic cannabinoid (SC) exclusion criteria, demographic characteristics, mental health status, resource utilization, and outcomes. CT indicates computed tomography; ED, emergency department; ICU, intensive care unit; NYC, New York City; PCP, phenylcyclidine.

Of the 42 cases reviewed, SC use was historically confirmed in 30 patients (24 men [80%]), with a mean age of 41 years (range, 21-59 years). Thirteen patients were undomiciled. Twenty-three patients were admitted to the ICU, whereas 7 patients received critical care services in the emergency department (ED). Twenty-five patients had a history of polysubstance abuse, psychiatric illness, or known personality disorder. Twenty patients admitted to smoking K2, and for 10 additional patients, K2 use was witnessed and recorded by friends, bystanders, or paramedics. Seven patients reported that they obtained K2 at main train stations in New York or after they were released from jail. Figure 1 summarizes the demographic characteristics, resource utilization, and clinical outcomes of the patients.

The ICU admissions were all for neurologic toxic effects and/or associated respiratory failure. There was no response to naloxone for 10 of 14 unresponsive patients (71%) with methadone or opioid coingestion. The primary neurologic presentations were coma (10 patients [33%]), agitation (10 patients [33%]), and seizure (6 patients [20%]). Among patients with extreme agitation, 2 (20%) required large amounts of sedatives to prevent self-harm and required intubation; 5 of 30 patients (16%) experienced traumatic body injuries after using K2.

Eighteen patients (60%) had acute respiratory failure, including 12 patients (40%) with hypercapnic respiratory failure, 3 (10%) with aspiration pneumonia, and 3 patients (10%) with acute respiratory distress syndrome (ARDS). Intubation was required for 21 patients (70%) for either acute respiratory failure or for airway protection, including 12 (40%) who presented with hypercarbia. The duration of intubation was less than 48 hours for 13 patients (52%), and 2 patients self-extubated. Eight patients (26%) presented with acute kidney failure. Table 1 summarizes the clinical findings of the critically ill patients.

**Table 1.** Clinical Findings and Outcomes in Patients With Reported Synthetic Cannabinoid Use and Intensive Care Unit Admission

Findings and outcomes	Patients, No. (%) (N = 30)^a^
Nervous system	
Seizure	6 (20)
Agitation	10 (33)
Coma or unresponsiveness	10 (33)
Pulmonary	
Hypoxemic respiratory failure or acute respiratory distress syndrome	3 (10)
Hypercarbia	12 (40)
Aspiration pneumonia	3 (10)
Cardiovascular	
ST-segment–elevation myocardial infarction or non–ST-segment–elevation myocardial infarction	2 (6)
Bradycardia	5 (16)
QT prolongation	9 (30)
Hypertensive emergency	2 (6)
Cardiac arrest	1 (3)
Acute renal failure or rhabdomyolysis	8 (26)
Traumatic body injuries	5 (16)
Invasive mechanical ventilation	21 (70)
For <48 h (n = 21)	13 (62)
Noninvasive ventilation	4 (12)
Hemodynamics	
Inotropes	3 (10)
Vasopressors	3 (10)
Advanced diagnostics	
Computed tomography	
Head	26 (86)
Spine	5 (16)
Lumbar puncture	1 (3)
Cardiac catheterization	2 (6)
Electroencephalogram	6 (20)
Urine toxicology studies	
No coingestions	16 (53)^b^
Coingestions	14 (46)
No response to naloxone (n = 14)	10 (71)
Ethyl alcohol	3 (10)
Outcomes	
ICU length of stay, d	
≤1	14
≤2	18
≥3	9
Discharged against medical advice or elopement	16 (53)
Psychiatric admission or rehabilitation	4 (13)
Death	1 (3)
Readmission associated with K2	5 (16)

^a^ Percentages do not sum to 100% because the patients had more than 1 sequela.

^b^ Three patients received benzodiazepines in the emergency department, urine toxicology was collected with delay, and were considered negative.

**Figure 2.**
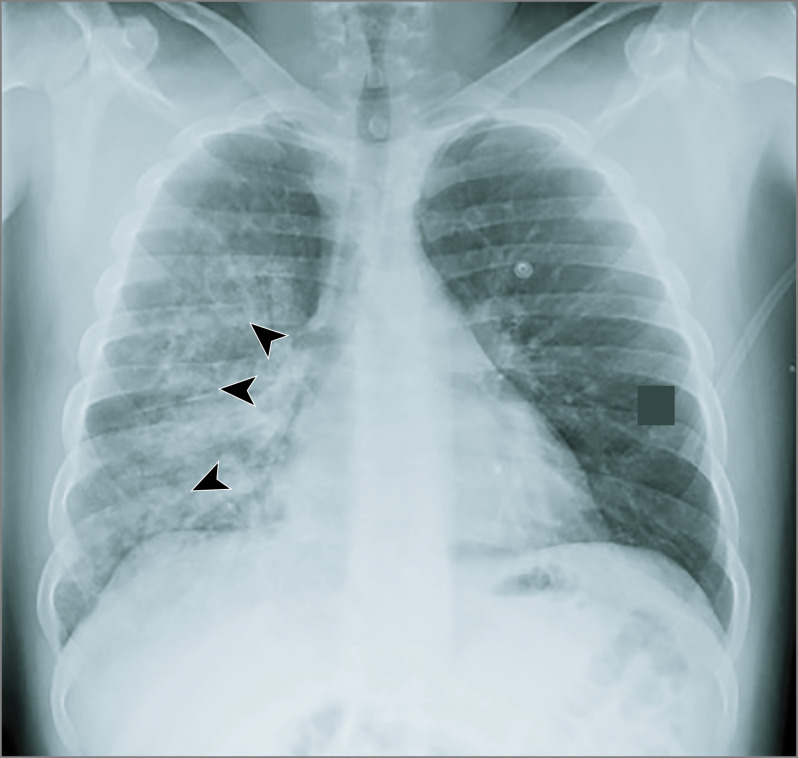
Chest Radiograph Obtained at Admission of Patient With Hypoxic Respiratory Failure and Hemoptysis Unilateral pulmonary edema (arrowheads) is noted in the clinical context of hemoptysis. Bronchoalveolar lavage findings were consistent with diffuse alveolar hemorrhage. The patient was mechanically ventilated for less than 10 hours. He left against medical advice shortly after his extubation (patient 30 in Table 2).

One young woman with history of asthma died of complications from ARDS shortly after presentation to the ED (patient 26 in Table 2). A man in his 30s presented with hemoptysis after smoking K2 and was found to have radiographically unilateral pulmonary edema (Figure 2). Bronchoscopy was performed before his discharge, and the findings were consistent with diffuse alveolar hemorrhage (patient 30 in Table 2). A man in his 50s was admitted with unresponsiveness and moderate ARDS after admitted K2 use. The ARDS resolved after 7 days of mechanical ventilation (patient 9 in Table 2). A man in his 20s with history of end-stage renal disease (ESRD) and pulmonary hypertension, as well as bipolar disorder and long-term marijuana use, was admitted with respiratory distress and was found to have anterolateral wall ST-segment–elevation myocardial infarction after smoking K2 for the first time. His care was complicated by elopement twice (patient 15 in Table 2). Our cohort included another patient with ESRD and coronary artery disease who presented with pulmonary edema shortly after K2 use (patient 29 in Table 2).

**Table 2.** Clinical Presentation in the Synthetic Cannabinoid Users of Our Cohort Focusing on Neurologic and Respiratory Manifestations

Patient No.	Age, decade/sex	Initial presentation	Coingestion	Urine toxicology findings	CT head findings	Ventilatory mode
1	50s/F	AMS, coma, seizure, hypercapnic respiratory failure	None	Negative	Normal	MV <1 d, self-extubated
2	20s/M	AMS, agitation	None	Negative	Normal	MV <1 d
3	50s/M	AMS, coma, acute respiratory failure	Ethyl alcohol	Cannabinoid benzodiazepine^a^	Normal	MV <1 d
4	40s/M	AMS, coma	Long-term methadone	Methadone	Normal	NIV
5	40s/M	AMS, coma	None	Negative	Normal	MV <2 d
6	40s/F	AMS, coma hypercapnic respiratory failure	Marijuana	Cannabinoid	NA	NIV
7	30s/M	AMS, agitation	None	Benzodiazepine^a^	Normal	MV <2 d
8	50s/M	AMS, coma	None	None	NA	NIV
9	50s/M	AMS, coma, respiratory failure	None	Negative	Frontal encephalomalacia	MV 7 d
10	30s/M	AMS, agitation	None	Negative	NA	NA
11	50s/M	AMS, seizure	None	Negative	Normal	MV <2 d
12	30s/F	AMS with nuchal rigidity	None	Negative	Normal	MV <1 d, self-extubated
13	30s/M	AMS, hypoxic respiratory failure	Cocaine	Cocaine	Normal	MV 9 d
14	30s/M	AMS, coma, hypercapnic respiratory failure	Opiates	Opiates	Normal	MV 4 d
15	20s/M	Mania	Marijuana	Cannabinoids	Normal	NA
16	30s/M	AMS, seizures, trauma code	Marijuana	Cannabinoids	Loss of gray white matter differentiation on CT head- reversible	MV <4 d
17	30s/F	AMS, coma	None	None	Normal	NIV
18	30s/M	AMS, agitation	Ethyl alcohol	Cocaine	Normal	MV
19	50s/M	AMS, seizures	None	None	Normal	MV 9 d
20	30s/M	AMS, extreme agitation	Long-term methadone	Methadone	Normal	MV <1 d
21	30s/M	AMS, extreme agitation, trauma code	None	Opiates^a^	Normal	NC
22	20s/M	AMS, agitation	None	None	Normal	MV
23	40s/M	AMS, agitation, trauma code	Long-term methadone	Methadone	Normal	MV 4 d
24	50s/M	AMS, agitation, trauma code	None	Benzodiazepine^a^	Normal	NA
25	40s/F	AMS, seizures	Long-term methadone	Methadone, benzodiazepine^a^	Normal	MV <1 d
26	20s/F	Hypoxic respiratory failure, acute respiratory distress syndrome, seizures	Bupropion, alprazolam	Benzodiazepine^a^	Normal	MV <1 d
27	30s/M	AMS, agitation, trauma code	Ethyl alcohol	Cannabis	Normal	MV <2 d
28	50s/M	AMS, coma	None	Cannabis, phenylcyclidine	Normal	MV <1 d
29	50s/M	Hypoxic respiratory failure, hypertensive emergency	None	Negative	Normal	Nasal canula
30	30s/M	Hypoxic respiratory failure, hemoptysis	None	Benzodiazepine^a^	Normal	MV <1 d

Abbreviations: AMS, altered mental status; CT, computed tomography; F, female; M, male; MV, mechanical ventilation; NA, not applicable; NIV, noninvasive ventilation.

^a^ Substances (benzodiazepines, opiates) given in the emergency department, collection of urine specimen obtained with delays.

**Figure 3.**
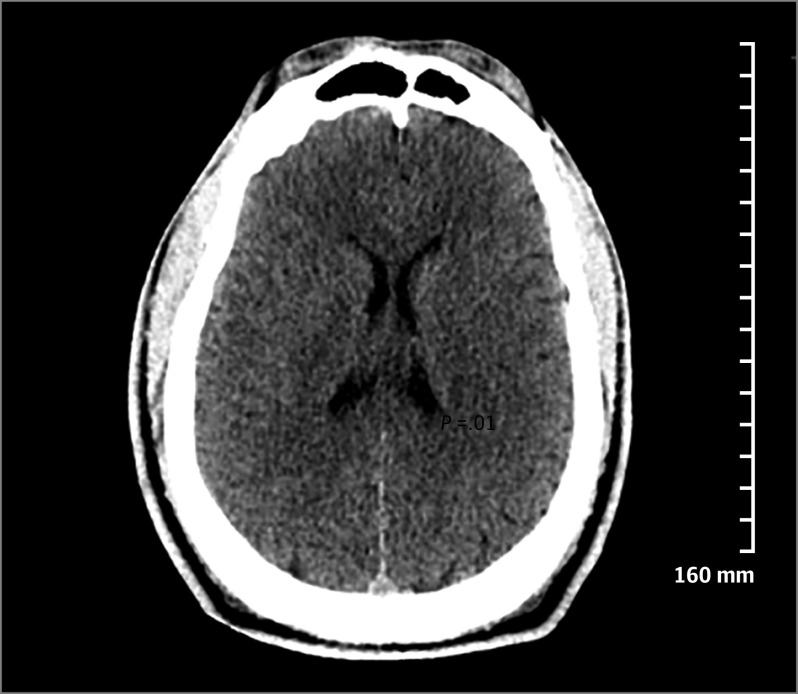
Computed Tomography Image of Patient With Altered Mental Status and Seizure Computed tomography of head was obtained without intravenous contrast agent. Note the diffuse cerebral edema and loss of gray-white differentiation. The patient self-extubated within 3 days after his presentation (patient 16 in Table 2).

Computed tomography scan of the head was performed for 26 patients (86%), and 6 patients (20%) underwent electroencephalogram monitoring (Table 1). One patient was found unresponsive, hypothermic, and hypoxemic on the street. He had absent brain stem reflexes and decerebrate posturing (patient 16 in Table 2). Computed tomography of the head showed global cerebral edema with loss of gray-white differentiation (Figure 3), consistent with anoxic brain injury; however, he improved and was extubated 40 hours later after treatment with mannitol and hypertonic saline. Lumbar puncture needed to be performed for a woman who presented unresponsive and with nuchal rigidity (patient 12 in Table 2). Two patients underwent diagnostic cardiac catheterization for chest pain with associated electrocardiographic abnormalities (patients 15 and 28 in Table 2).

All patients underwent routine serum and urine toxicology testing, which was negative in 16 cases; however, testing revealed at least 1 coingestion (cocaine, marijuana, alcohol, or methadone) in the other 14 cases. We included in our cohort 2 patients who admitted to long-term cocaine use but who reported their last use days before their presentation in the ED (patients 13, 18 Table 2), and a patient whose urine toxicology findings indicated phenylcyclidine; he denied using phenylcyclidine but admitted to K2 use (patient 28, Table 2). Five patients in our cohort were known K2 users with K2-related ED visits in our hospital system noted days before their admission that required critical care attention (patients 1, 5, 9, 10, and 19 in Table 2).

Rhabdomyolysis was noted in 8 patients (26%) (Figure 1). QT prolongation, which has been reported in the literature^17^ in association with SC use, was noted in 10 patients (33%). Sixteen patients (53%) left the hospital against medical advice, and 4 were admitted in the psychiatric unit after the resolution of the acute effects of intoxication for persistence of the behavioral alteration and the unsafety of community disposition (patients 9, 10, 16, and 23 in Table 2).

## Discussion

Synthetic cannabinoids were developed in research in the 1970s to study the cannabinoid system.^6,18^ It was not until 2008 that SCs reemerged in the US as a cheap recreational drug.^1,19^ The harmful effects of exposure to SC were first reported in the US in 2009.^3^ Cannabinoid CB1 receptors are among the most abundant receptors expressed in the brain and play a significant role in the modulation of GABA and glutamate neurotransmission,^10,20^ affecting the neuronal functioning of the prefrontal cortex^21^ and, thus, emotional processing, sensory perception, and elaboration of incoming sensory information.^12^ Other serious adverse effects, particularly sympathomimetic and hallucinogenic effects related to new compounds, may be due to indirect activation of other receptors via excess activation of cannabinoids receptors, direct receptor activations due to mixed receptor effects of new cannabinoids, or possibly adulterants, including plant material effects^7^.

Synthetic cannabinoids are often smoked.^11^ When inhaled, effects begin within minutes, with a shorter duration of action and quicker time to peak onset compared with nonsynthetic cannabinoids.^22^ Intoxication with SCs may manifest as violent behavior,^6^ psychosis,^23^ paranoia,^10,23^ delirium,^23^ and withdrawal.^10,13^ Patients may develop severe neurologic toxic effects, including generalized tonic-clonic seizures,^10,24^ cardiovascular events, including myocardial infarction,^25,26^ transient cerebral edema mimicking anoxic brain injury,^27^ intracranial hemorrhage, and cerebral ischemic events.^28^ Pulmonary manifestations include diffuse alveolar hemorrhage,^29^ respiratory failure, with radiographs showing diffuse pulmonary infiltrates and tree-in-bud morphologic features, and computed tomography findings mimicking organizing pneumonia in young users.^30^ Severe rhabdomyolysis, acute kidney injury with acute tubular necrosis,^6,31,32^ and death are also manifestations of SC intoxication.^10,11,19^

In our series, all 30 patients required critical care interventions, with 23 requiring ICU level of care. Most of the patients were young men with a history of either psychiatric disorders or substance abuse. Most presented with mental status changes ranging from coma (10 patients), to agitation (10 patients), to seizures (6 patients). Bizarre behavior was documented in some patients without further characterization. Twenty-one patients required invasive mechanical ventilation, including 40% who presented with hypercarbia. Patients also presented with acute kidney injury (26%) and cardiac toxic effects, including myocardial infarction, bradycardia, and QT prolongation. Although they were critically ill at admission, rapid improvement was the usual course, and most (53%) left the hospital against medical advice.

In our experience, catastrophic presentations of K2 intoxication, such as cerebral edema and ARDS, have the potential for rapid reversal. We have previously described 1 patient who was found unresponsive, hypothermic, and hypoxemic on the street.^27^ He had absent brain stem reflexes and decerebrate posturing (patient 16 in Table 2). Computed tomography of the head showed global cerebral edema with loss of gray-white differentiation (Figure 3), consistent with anoxic brain injury; however, he improved and was extubated 40 hours later after treatment with mannitol and hypertonic saline. A man in his 50s was admitted with unresponsiveness and moderate ARDS after admitted K2 use. The ARDS resolved after 7 days of mechanical ventilation (patient 9 in Table 2). A woman in her 20s with a history of asthma and anxiety, presented with respiratory distress, altered sensorium, and generalized tonic-clonic seizures after K2 inhalation. She was profoundly hypoxemic upon presentation and had repeated cardiac arrests (patient 26 in Table 2). She eventually died despite aggressive resuscitative efforts.

What remains unclear is whether these manifestations of K2 intoxication are due to the toxicity of the chemicals or due to excessive dosages.^5,7^ The amount of K2 smoked could not be quantified, but a few patients reported that they smoked only 1 cigarette. We are reluctant to comment on the extent of effect when patients are found with coingestions, but for those with opioid coingestion, we observed that some patients had no response to naloxone. On the basis of our experience, we have a high index of suspicion for SC intoxication in cases of severe and uncontrolled agitation in the setting of negative or inconsistent toxicology studies. Similarly, we suspect SC use in cases of unresponsiveness with no response to naloxone, or with an atypical presentation of known intoxication. Our cohort included 2 patients with significant comorbidity, including 1 with ESRD and pulmonary hypertension (patient 15 in Table 2) and 1 with ESRD and coronary artery disease (patient 29 in Table 2). We do not conclude that the SC was an isolated causative factor for their decompensation. Yet, because of the observed temporal association between SC smoking and when they sought and received medical attention, we speculate that the acute cardiovascular effects of SC use led to their decompensation.

Understanding the limitations of toxicology screening is important. Although detection of some SCs is possible with means of liquid chromatography or mass spectrometry in specialized laboratories,^33^ conventional drug test panels available in most hospitals do not detect the broad range of SCs. Metabolites of SC can be detected by specialized forensic toxicology laboratories.^5,7^ Efforts to identify other ever-emerging SC metabolites continue. During an outbreak in Brooklyn, New York, in 2016,^16^ the substance AMB-FUBINACA (methyl 2-[1-{4-fluorobenzyl}-1*H*-indazole-3-carboxamido]-3-methylbutanoate) was isolated in 8 of 18 patients who used the SC AK-47 24 Karat Gold. Similarly, the molecule ADB-PINACA (N-[1-amino-3,3-dimethy-1-oxobutan-2-yl]-1-pentyl-1H-indazole-3-carboxamide) was identified during an outbreak in Colorado, where patients presented mainly with neurologic and cardiac symptoms; however, only 7 of 76 patients who presented to the ED required ICU level of care in that series.^24^ Hence, it is important going forward that when SC intoxication is suspected, public health officials should work closely with toxicologists and hospital staff to obtain serum samples and perform comprehensive testing for various toxins.

### Limitations

This study has several limitations, including the retrospective nature of the analysis. Our cases were confirmed by history and emergency medical services report and not by chemical analysis, which was not available at our institution. Because signs of SC intoxication usually abate over time, it is possible that cases were missed. The toxicology studies sent from our ED and ICU reflected the presence of other coingestions. We excluded cases where phenylcyclidine, cocaine, amphetamines, or high alcohol levels were isolated in the urine or serum studies. We included in our cohort 2 patients who admitted to long-term cocaine use, but who reported last use days before their presentation in the ED (patients 13 and 18 in Table 2), and a patient whose urine toxicology findings indicated phenylcyclidine; he denied its use but admitted to K2 use (patient 28 in Table 2). Given the difficulty in identifying SC as an intoxicant, it is inevitable that SC use is both underreported and underdiagnosed.^11,34^ Most patients who admitted to SC use did so only when they were specifically asked; hence, this series could be an underestimate of the true prevalence of critically ill patients with SC intoxication. Five patients in our cohort were known K2 users with K2-related ED visits in our hospital system noted days before their admission that required critical care attention (patients 1, 5, 9, 10, and 19 in Table 2).

## Conclusions

Health care practitioners should be familiar with the potential adverse effects of SC and suspect its use in the at-risk population. Despite the toxicity of SC and the legal acts to stop its distribution, abuse is expected to increase, in part because of the ease of acquisition though online retailers and a false perception among users that SCs are safe, affordable alternatives to cannabis. Management of SC intoxication in the critically ill patient remains supportive, targeting hemodynamic stabilization, electrolyte balance, seizure control, hydration with intravenous fluids, and observation until the patient demonstrates clinical improvement. Inability to control symptoms or agitation or seizures despite escalating doses of benzodiazepines should alert the physician for the potential need for tracheal intubation. Even nearly fatal cases appear to have the potential of reversibility.

To our knowledge, this is the largest case series of critically ill patients with SC intoxication who required care in the ICU. These patients presented with a variety of clinical symptoms and outcomes. Given the increase in SC use, its low cost, and the ease with which it can be obtained, SC use appears to be a continued public health threat. This series illustrates the level of critical illness that can occur with SC use and helps identify and characterize the health risks associated with SC exposure.
